# Eating disorder risks and psychopathological distress in Italian high school adolescents

**DOI:** 10.1186/s13052-024-01717-7

**Published:** 2024-08-07

**Authors:** Valeria Calcaterra, Vittoria Carlotta Magenes, Martina Basso, Veronica Conte, Giulia Maggioni, Susanna Russo, Annalisa De Silvestri, Valentina Fabiano, Elisabetta Agnese Marrocco, Pierangelo Veggiotti, Gianvincenzo Zuccotti

**Affiliations:** 1 Department of Internal Medicine and Therapeutics, University of Pavia, Pavia, 27100 Italy; 2 Pediatric Department, Vittore Buzzi Children’s Hospital, Milan, 20154 Italy; 3 Child and Adolescent Neuropsychiatry Unit (UONPIA), ASST-Fatebenefratelli-Sacco, Milan, 20154 Italy; 4 Biometry & Clinical Epidemiology, Scientific Direction, Fondazione IRCCS Policlinico San Matteo, Pavia, 27100 Italy; 5 Department of Biomedical and Clinical Sciences, University of Milan, Milano, 20157 Italy; 6 Pediatric Neurology Unit, Buzzi Children’s Hospital, Milan, 20154 Italy

**Keywords:** Eating disorder, Psychopathological distress, Adolescents, School, Screening

## Abstract

**Background:**

Psychopathological disorders are often comorbid diagnosis in eating disorders (EDs). We aimed to assess the presence of psychopathological traits and symptoms associated with EDs in an Italian high school adolescent population.

**Methods:**

A sample of high school adolescents was enrolled, and demographic and clinical data were collected. Two self-report questionnaires, the Eating Disorder Inventory-3 (EDI-3) and the Questionnaire for the Assessment of Psychopathology in Adolescence (Q-PAD), were administered.

**Results:**

548 adolescents (333 F/215 M; 16.89 ± 0.85 years) were included. Symptoms associated with EDs of clinical or high clinical concern were prevalent in a range of individuals, with percentages varying from 26.82% for body dissatisfaction to 51.83% for Interoceptive Deficits. The findings from the Q-PAD assessment indicated the presence of psychological distress, leading to discomfort or challenging situations requiring potential intervention in a percentage of adolescents ranging from 2.93% for psychosocial risks to 23.77% for anxiety. These percentages showed differences between genders (F > M, *p* < 0.001). Our study also highlighted an association between symptoms of EDs and lifestyle factors within families. We observed correlations between Q-PAD measures and EDI-3 scores, including a positive correlation between Q-PAD and EDI-3 body dissatisfaction (*r* = 0.7), Q-PAD interpersonal conflicts and EDI-3 interpersonal problems (*r* = 0.6) and a negative correlation between Q-PAD self-esteem and well-being and EDI-3 ineffectiveness Composite (*r*=-0.7).

**Conclusions:**

a substantial prevalence of ED symptoms and psychological distress among high school adolescents were recorded. These conditions are interrelated, suggesting the importance of addressing them comprehensively. Early detection is essential to improve treatment outcomes and to implement preventive strategies.

**Supplementary Information:**

The online version contains supplementary material available at 10.1186/s13052-024-01717-7.

## Background

Adolescence represents a critical phase characterized by significant socio-affective and neurocognitive transformations, accompanied by an elevated susceptibility to mental health issues and disorders [1, 2]. Of particular concern are anxiety disorders and depression, which rank among the most common mental health disorders during adolescence [3]. Notably, these conditions are often comorbid diagnosis in eating disorders (EDs), especially within this age group [4, 5]. EDs are now among the most prevalent chronic disorders in adolescents and young adults, with a noticeable increase in prevalence among younger children [6, 7].

Although these disorders predominantly affect females, recent years have seen a rising prevalence among males and minority populations. Core symptoms of EDs revolve around abnormal eating or weight-control behaviors [8–10]. Alarmingly, despite their high prevalence, EDs frequently remain underdiagnosed, leading to a protracted and severe course [10–12], especially when accompanied by other comorbid conditions [2]. A recent systematic review and meta-analysis by López-Gil et al. analyzed thirty-two studies encompassing 63,181 participants from 16 countries, revealing that 22% of children and adolescents exhibited disordered eating behaviors, with even higher proportions among girls [13]. Adolescents are at an increased risk of developing EDs, which is strongly associated with body dissatisfaction and body image concerns [4, 5], particularly among females. Furthermore, depressive disorders have been on the rise among adolescents, especially girls, worldwide, from Finland [14] to the United States [15], the UK [16], and Europe [2, 17].

Crucially, anxiety disorders and depression are intricately linked to more severe ED psychopathology [18]. Comorbid depression and anxiety symptoms in individuals with EDs signify heightened symptom severity and a less favorable prognosis, particularly among young females [2]. However, research examining the relationship between anxiety, depression, and ED symptoms in young adults is limited. Theoretical models postulate a shared etiology among anxiety, depression, and EDs [19], with disordered eating behaviors often viewed as maladaptive strategies for regulating negative emotional states [19]. For instance, high levels of anxiety can lead to dysfunctional emotion regulation strategies such as binge-eating, potentially exacerbating eating disorder psychopathology, and vice versa [2, 20]. In Fairburn et al.‘s transdiagnostic theory of EDs, low self-esteem and perfectionism play crucial roles as maintaining factors [21]. Eating disorder symptoms can further erode self-esteem and provoke concerns about negative social evaluation (e.g., fear of being negatively judged by others) [22]. This low self-esteem, in turn, is associated with various adverse health outcomes and is itself a risk factor for the development of depression [22, 23]. Importantly, perfectionism, another key factor in EDs [21], is also found at elevated levels in anxiety disorders and depression [2].

Simultaneously, alongside the rising prevalence of depressive disorders and EDs, pediatric obesity has emerged as a serious global health concern [24–26]. The pathogenic mechanisms of obesity are multifactorial, involving complex interactions between genetic, epigenetic, environmental, physiological, and sociocultural factors [27]. Obesity carries significant comorbidities that adversely affect psychosocial well-being and overall quality of life [28, 29]. While obesity is not classified as an eating disorder per se, it is closely intertwined with EDs and can be viewed as part of a continuum, with one condition often leading to the other, such as in binge-eating disorder and bulimia nervosa. These conditions share similar psychosocial, metabolic, and nutritional health consequences [30–33]. Various mechanisms connecting obesity with EDs and vice versa have been proposed, encompassing environmental factors (e.g., family and peer teasing, perceived social pressure, bullying, or criticism) and individual risk factors (e.g., genetic predisposition, negative self-evaluation, low self-esteem, and body dissatisfaction) [33].

Given the high prevalence of EDs in adolescents, it is paramount to investigate the interplay between psychological distress and EDs. The primary objective of this study is to illustrate a context of EDs in an Italian high school adolescent population, examining the presence and intensity of psychopathological traits and symptoms associated with EDs, and evaluating psychopathology. Additionally, the study will record lifestyle behaviors. Our hypothesis posits that there is an underreported prevalence of psychopathological traits related to eating and psychopathological domains, which coexist. Identifying at-risk individuals early on can facilitate the implementation of preventive strategies and contribute to addressing this issue, ultimately improving public health.

## Methods

### Participants

Between March and June 2023, we enrolled a targeted sample comprising students from three high schools in the province of Milan, Italy. The schools enrolled were two scientific high schools and one scientific high school with applied sciences option and the individuals were selected based on their availability and willingness to take part to the project. Before enrolling the individuals, our project was explained to the school headmaster, the faculty and the school board.

To meet the inclusion criteria, participants had to be aged 15–18, Italian speakers, and both male and female individuals were eligible. Individuals with confirmed physical and mental disorders were not excluded in our study. The study received approval from the institutional ethics committee Milano Area 1 (protocol number 0017683/2023, experimental number n.2023/ST/003) and adhered to the principles of the 1975 Declaration of Helsinki, as revised in 2008. Specifically, individuals over 18 years gave their written consent to participate; instead, for individuals under 18 years both legal guardians’ written consent an assent by adolescents themselves were collected.

### Participant information

We collected demographic and clinical data from all participants, including age, gender, weight (in kilograms), height (in meters), and calculated body mass index (BMI) by dividing weight in kilograms by the square of height in meters. Age- and sex-adjusted BMI was calculated using World Health Organization Growth Standards. We used BMI values below the ≤ 2nd percentile to identify underweight and those above the ≥ 85th percentile for overweight or obesity. A questionnaire (Table 1) was distributed to assess health habits, physical activity, vital signs, sleep patterns, food frequency, eating habits, and meal planning. We also considered parental socio-demographic characteristics, family history of EDs, and any previous psychological treatments (Table 1). To assess psychopathology, we administered two self-report questionnaires, the Eating Disorder Inventory-3 (EDI-3), and the Questionnaire for the Assessment of Psychopathology in Adolescence (Q-PAD).

**Table 1:** Data on lifestyle behaviors and family features

Features	Percentage of cases	*P* females vs. males
**Lifestyle behaviors**		
**Eating habits**		
Breakfast Never Rarely Always Often	7.13% (F 64.10%; M 35.90%) 24.50% (F 72.39%; M 27.61%) 49.91% (F 51.65%; M 48.35%) 18.46% (F 68.32%; M 31.68%)	< 0.001
Breakfast drink Chocolate Milk/yogurt /cappuccino Juice fruit/orange juice Tea/caffè	0.48% (F 100%; M 0%) 54.25% (F 54.48%; M 45.52%) 13.36% (F 63.64%; M 36.36%) 31.98% (F 68.99%; M 31.01%)	0.01
Breakfast food Biscuits/biscuit slice /snacks Fruit Pizza/focaccia/toast Cured meals/cheeses	91.07% (F 59.26%; M 40.74%) 5.56% (F 78.57%; M 21.43%) 1.98% (F 60.0%; M 40.0%) 1.39% (F 42.86%; M 57.14%)	0.17
Three regular meals (breakfast/lunch/dinner) Never Rarely Always Often	3.13% (F 70.59%; M 29.41%) 14.73%(F 71.25%; M 28.75%) 54.70% (F 51.52%; M 48.48%) 27.44% (F 72.48%; M 27.52%)	< 0.001
Drinks between meals Water Sugar-sweetened beverages Beer/vine Juice fruit/orange juice	66.91% (F 57.42%; M 42.58%) 23.16% (F 66.67%; M 33.33%) 1.65% (F 55.56%; M 44.44%) 8.27 (F 71.11%; M 28.89%)	0.12
Fruit/day Never Rarely Always Often	11.13% (F 70.49%; M 29.51%) 39.05% (F 62.15%; M 37.85%) 12.59% (F 52.17%; M 47.83%) 37.23% (F 59.31%; M 40.69%)	0.17
Vegetables/day Never Rarely Always Often	9.89% (F 64.81%; M 35.19%) 25.27% (F 56.52%; M 43.48%) 26.92% (F 62.59%; M 37.41%) 37.91% (F 61.35%; M 38.65%)	0.64
Dessert/day Never Rarely Always Often	12.82% (F 57.14%; M 42.86%) 50.37% (F 60.73%; M 39.27%) 7.33% (F 50.0%; M 50.0%) 29.49% (F 65.22%; M 34.78%)	0.29
Beer/vine with meals Never Rarely Always Often	67.10% (F 66.03%; M 33.97%) 29.41% (F 50.62%; M 49.38%) 0.74% (F 25.0%; M 75.0%) 2.76% (F 46.67%; M 53.33%)	0.02
Milk-yogurt/day Never Rarely Always Often	17.25% (F 73.40%; M 26.60%) 28.44% (F 67.74%; M 32.26%) 27.71% (F 45.03%; M 54.97%) 26.61% (F 62.07%; M 37.93%)	< 0.001
Water during the day Never Rarely Always Often	6.25% (F 100%; M 0%) 20.77% (F 76.99%; M 23.01%) 39.71% (F 43.52%; M 56.48%) 33.27% (F 64.09%; M 35.91%)	< 0.001
**Physical activity level**		
Physical activity/year Never Always/all year round Only a few season Sometimes	7.71% (F 73.81%; M 26.19%) 62.57% (F 53.08%; M 46.92%) 14.31% (F 69.23%; M 30.77%) 15.41% (F 77.38%; M 22.62%)	< 0.001
Physical activity/ week 1–2 h 3–4 h > 4 h None	18.43% (F 70.30%; M 29.70%) 19.53% (F 72.90%; M 27.10%) 42.7% (F 41.88%; M 58.12%) 19.34% (F 81.13%; M 18.87%)	< 0.001
Free time activities TV/music/computer/lecture Walks Shopping Sport	55.51% (F 60.93%; M 39.07%) 15.25% (F 74.70%; M 25.38%) 10.48% (F 92.98%; M 7.02%) 18.75% (F 31.37%; M 68.63%)	< 0.001
Computer/TV 1–2 h 3–4 h 5–6 h > 6 h	51.29% (F 62.95%; M 37.05%) 34.13% (F 58.38%; M 41.62%) 11.62% (F 57.14%; M 42.68%) 2.95% (F 68.75%; M 31.25%)	0.62
Physical activity at school It bores you It makes you feel better It tires you It stimolates you	50.74% (F 69.57%; M 30.43%) 28.12% (F 44.44%; M 55.56%) 8.82% (F 79.17%; M 20.83%) 12.32% (F 49.25%; M 50.75%)	< 0.001
Lifestyle Moderately active Very active Very sedentary Sedentary	53.65% (F 64.97%; M 35.03%) 22.99% (F 42.06%; M 57.94%) 1.82% (F 70.0%; M 30.0%) 21.53% (F 69.49%; M 30.51%)	< 0.001
**Sleep**		
*Hours each night* < 6 h < 8 h > 8 h	20.11% (F 71.38%; M 28.70%) 64.06% (F 53.78%; M 46.22%) 15.83% (F 75.29%; M 24.72%)	< 0.001
**Family features**		
**Features of the parents**	**Percentage of cases**	**P females vs. males**
Education levels Elementary school Middle school High school university	0.58% (F 100%; M 0%) 7.38% (F 60.53%; M 39.47%) 52.82% (F 61.76%; M 38.24%) 36.86% (F 57.43%; M 42.57%)	0.48
Socio-economic status High Low Middle-high Middle-low	0.81% (F 75.0%; M 25.0%) 1.43% (F 71.43%; M 28.57%) 60.90% (F 64.21%; M 35.79%) 36.86% (F 55.80%; M 44.20%)	0.26
Profession Unemployed Employee Businessman Freelance Worker Retired Other	1.37% (F 71.43%; M 28.57%) 50.78%(F 56.76%; M 43.24%) 6.47%(F 60.61%; M 39.39%) 14.31% (F 61.64%; M 38.36%) 6.86% (F 65.71%; M 34.29%) 0.59%(F 0%; M 100%) 19.61% (F 69.0%; M 31.0%)	0.12
Familiarity for eating disorders * Yes No	5.27% (F 58.97%; M 41.03%) 94.73% (F 85.19%; M 14.81%)	0.007
Previous psychopharmacological treatments No Si	94.86% (F 60.74%; M 39.26%) 5.14% (F 56.0%; M 44.0%)	0.63

F = females; M = males

All questionnaires and related variables are reported in Supplementary Material 1.

### Assessment of psychopathology

#### Eating disorder inventory-3 (EDI-3)

We administered the EDI-3 to participants to clinically evaluate the presence and intensity of psychopathological traits and symptomatology related to EDs. The EDI-3 comprises three eating disorder-specific scales (Drive for Thinness, Bulimia, and Body Dissatisfaction) and nine general psychological scales, relevant to but not specific to EDs (Low Self-Esteem, Personal Alienation, Interpersonal Insecurity, Interpersonal Alienation, Interoceptive Deficits, Emotional Dysregulation, Perfectionism, Asceticism, Maturity Fears). These 12 scales yield six composite scores, including a specific Eating Disorder Risk Composite and five general integrative psychological constructs (Ineffectiveness Composite, Interpersonal Problems Composite, Affective Problems Composite, Overcontrol Composite, and Global Psychological Maladjustment). In this study, we utilized a validated Italian version of the EDI-3 [34].

#### Questionnaire for the assessment of psychopathology in adolescence (Q-PAD)

Participants self-administered the Q-PAD to assess various psychopathological domains. The questionnaire comprises 81 items on a Likert-type scale ranging from 1 to 4. Scores for each item within a scale were summed, and the results were converted to percentile values. The Q-PAD generates eight scores corresponding to the following main psychological areas: body dissatisfaction, anxiety, depression, substance abuse, interpersonal conflicts, family problems, uncertainty about the future, and psychosocial risks. A ninth domain, focused on self-esteem and well-being, is assessed in positive terms.

### Statistical analysis

A minimum of 500 subjects were required can estimate prevalence of ED and distress with a good precision (at worst 4%). Quantitative variables were described using either mean ± standard deviation (SD) for continuous data, while qualitative variables were presented as counts or percentages as appropriate. The assumption of data normality was evaluated using the Shapiro-Wilk test. For the analysis of the association between categorical variables, Fisher’s exact test was employed, and comparisons between gender groups were conducted using the independent t-test. Correlational analyses, either Pearson or Spearman, were performed to explore potential relationships between psychological scales. A p-value less than 0.05 was considered statistically significant. All statistical analyses were conducted using Stata software version 16.1 (StataCorp USA).

## Results

### Clinical features

We enrolled a total of 548 adolescents (333 females and 215 males) with a mean age of 16.89 ± 0.85 years (16.9 ± 0.04 years for females vs. 16.8 ± 0.05 years for males, *p* = 0.8). The average BMI was 20.9 ± 2.9 kg/m², with no significant difference between sexes (females 20.90 ± 3.1 vs. males 20.90 ± 2.42; *p* = 0.8). Normal weight was observed in 89.9% of adolescents, while 1.1% were underweight, and 9.1% were overweight or obese. Notably, there was a higher prevalence of underweight (1.9% vs. 0%) and overweight/obesity (10.4% vs. 7.1%) in females compared to males (*p* = 0.05). No participants had a known diagnosis of disordered eating behaviors.

### Lifestyle behaviors

Table 1 presents the lifestyle behaviors of the enrolled adolescents. Notably, 31.63% of individuals reported rarely or never having breakfast. Irregular meals were noted in 17.86% of cases, and 23.16% reported consuming sugar-sweetened beverages between meals. Additionally, 50.18% rarely or never consumed fruits, while 35.16% reported the same for vegetables. Correct breakfast habits (*p* < 0.001), adherence to regular meals (*p* < 0.001), and adequate consumption of milk derivatives (*p* < 0.001) were more prevalent in males compared to females. On the contrary, the consumption of beer/wine with meals was more prevalent in males (*p* = 0.02). Water consumption during the day was more appropriately practiced by females compared to males (*p* < 0.001). No significant gender differences were observed in other eating habits (all *p* > 0.05).

Regarding physical activity, 23.35% of cases reported a sedentary lifestyle. Approximately 19.34% of adolescents did not engage in any form of physical activity during the week, and 14.57% used a computer/TV for more than 5 h per day. Males displayed a higher level of physical activity and differences in the type of free time activities compared to females (*p* < 0.001). A majority of the adolescents (84.17%) reported sleeping less than eight hours per night, with 20.11% sleeping less than 6 h. Correct sleep habits were more prevalent among females than males (*p* < 0.001). A significant correlation (*p* < 0.001) was observed between the presence of unhealthy habits and an altered psychopathological profile (at least 2 pathological Q-PAD scales).

### Family features

Table 1 provides information on socio-demographic characteristics, familiarity with EDs, and previous psychological treatments of parents. Parents showed high education levels (52.82% completed high school, and 36.36% attended university) and had a middle socio-economic status (60.90%), with no significant differences between the parents of female and male adolescents. Familiarity with EDs was recorded in 5.27% of cases, with a higher prevalence among female parents compared to male parents (*p* = 0.007).

### Psychopathological assessment

#### Eating disorder inventory-3 (EDI-3)

**Fig. 1:**
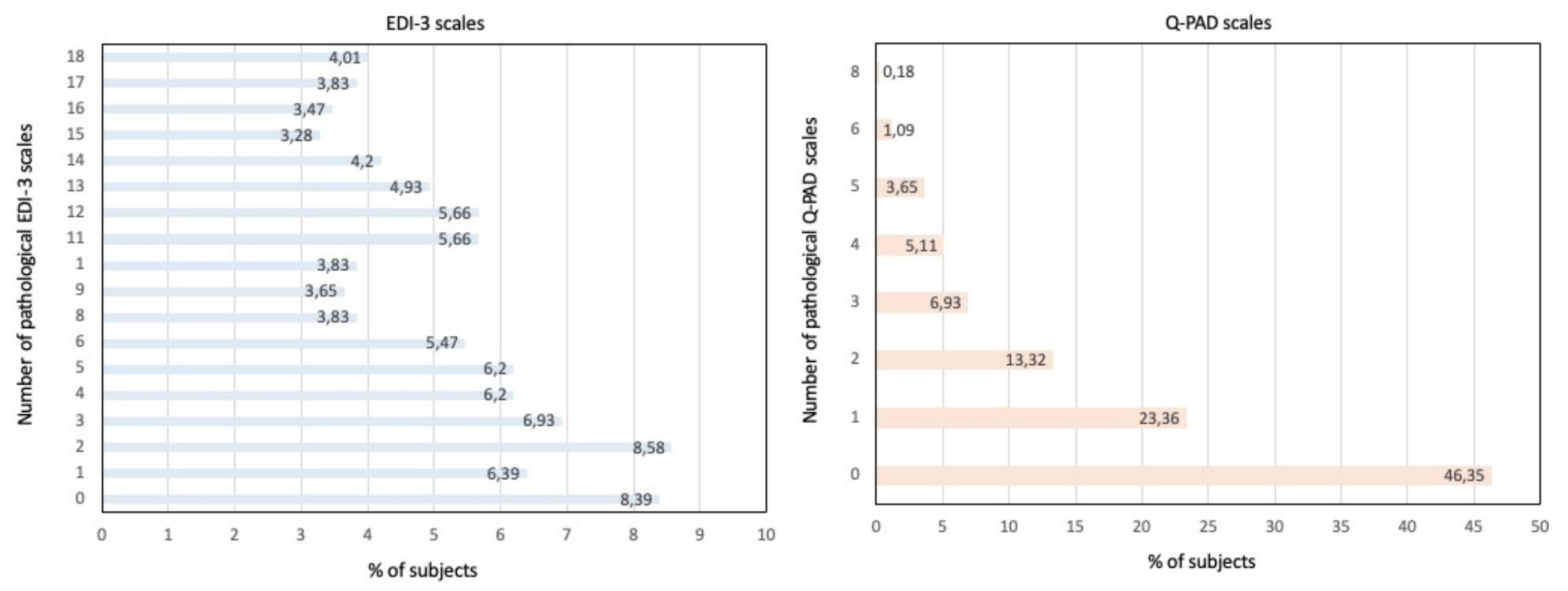
Percentages of subjects with one or more pathological EDI-3 (panel **A**) and Q-PAD (panel **B**) scales

In Fig. 1, Panel A, we present the percentages of individuals exhibiting one or more pathological EDI-3 scales. Table 2 provides the mean values, prevalence, and intensity of each EDI-3 scale and composite scale. Overall, we observed the presence of symptomatology associated with eating disorders, with clinical or high clinical interest scores, ranging from 26.82% for body dissatisfaction to 51.83% for the Interoceptive Deficits composite. Females displayed a higher prevalence of psychopathological traits associated with eating disorders, with greater intensity, in both eating disorder-specific scales and general psychological scales, in comparison to males (all *p* < 0.001). When considering different age groups (15-15.99 years, 16-16.99 years, 17-17.99 years, 18 years), no significant differences were noted in scale scores, except for the Interpersonal Problems composite (*p* = 0.02), which was more frequently present with clinical interest in the 16-16.99 years age group.

**Table 2:** Eating disorder Inventory-3 (EDI-3) scales and composites scale

Eating disorder-specific scales	Mean value ± standard deviation	*P* females vs. males	Score % of subjects (*n* = 548) (% of pathological subjects according to gender)				*p* females vs. males
			Low 1–24	Typical 25–66	Clinical interest 67–84	High clinical interest 85–99	
Drive for Thinness	42.14 ± 31.57 (F 53.83 ± 1.65; M 24.04 ± 1.66)	*p* < 0.001	35.48% (F 35.57%; M 64.43%)	35.58% (F 62.05%; M 37.95%)	19.16% (F 85.71%; M 14.29%)	9.85% (F 98.15%; M 1.85%)	< 0.001
Bulimia	55.16 ± 32.86 (F 61.14 ± 1.74; M 45.91 ± 2.21)	*p* < 0.001	19.53% (F 47.66%; M 52.34%)	29.01% (F 51.57%; M 48.43%)	30.66% (F 65.48%; M 34.52%)	20.80% (F 78.95%; M 21.05%)	< 0.001
Body Dissatisfaction	42.38 ± 28.87 (F 53.55 ± 1.49; M 25.08 ± 1.49)	*p* < 0.001	35.22% (F 35.23%; M 64.77%)	37.96% (F 63.46%; M 36.54%)	16.97% (F 90.32%; M 9.68%)	9.85% (F 90.74%; M 9.26%)	< 0.001
**General psychological scales**							
.Low Self-Esteem	50.82 ± 28.38 (F 59.23 ± 1.45; M 37.78 ± 1.77	*p* < 0.001	21.17% (F 34.48%; M 65.52%)	41.79% (F 56.77%; M 43.23%)	24.82% (F 77.94%; M 22.06%)	12.23% (F 85.07%; M 14.93%)	< 0.001
Personal Alienation	55.91 ± 31.75 (F 63.06 ± 1.77; M 44.85 ± 1.86)	*p* < 0.001	20.62% (F 45.13%; M 54.87%)	38.32% (F 53.33%; M 46.67%)	20.99% (F 66.09%; M 33.91%)	20.07% (F 85.45%; M 14.55%)	< 0.001
Interpersonal Insecurity	59.28 ± 28.30 (F 63.35 ± 1.56; M 52.79 ± 1.82)	*p* < 0.001	14.08% (F 54.55%; M 45.45%)	37.84% (F 51.21%; M 48.79%)	26.14% (F 63.64%; M 36.36%)	21.94% (F 77.50%; M 22.50%)	< 0.001
Interpersonal Alienation	51.34 ± 29.79 (F 57.24 ± 1.62; M 42.2 ± 1.88)	*p* < 0.001	22.81% (F 48.80%; M 51.20%)	43.07% (F 54.24%; M 45.76%)	16.24% (F 71.91%; M 28.09%)	17.88% (F 81.63%; M 18.37%)	< 0.001
Interoceptive Deficits	62.64 ± 27.57 (F 71.27 ± 1.37; M 49.28 ± 1.76)	*p* < 0.001	12.41% (F 33.82%; M 66.18%)	35.77% (F 44.90%; M 55.10%)	23.18% (F 70.87%; M 29.13%)	28.65% (F 84.08%; M 15.92%)	< 0.001
Emotional Dysregulation	58.43 ± 31.28 (F 66.89 ± 1.58; M 45.32 ± 2.06)	*p* < 0.001	16.24% (F 39.33%; M 60.67%)	35.77% (F 50.51%; M 49.49%)	21.17% (F 63.79%; M 36.21%)	26.82% (F 85.03%; M 14.97%)	< 0.001
Maturity fears	56.06 ± 28.96 (F 62.93 ± 1.54; M 50.04 ± 2.01)	*p* < 0.001	16.97% (F 45.16%; M 54.84%)	37.04% (F 55.17%; M 44.83%)	21.17% (F 70.69%; M 29.31%)	24.82% (F 71.32%; M 28.68%)	< 0.001
Perfectionism	56.06 ± 1.23 (F 60.60 ± 1.58; M 49.03 ± 1.89)	*p* < 0.001	17.52% (F 46.88%; M 53.12%)	39.85% (F 55.14%; M 44.86%)	24.09% (F 66.67%; M 33.33%)	19.34% (F 77.36%; M 22.64%)	< 0.001
Ascetism	58.71 ± 27.49 (F 63.70 ± 1.48; M 50.98 ± 1.93)	*p* < 0.001	14.68 (F 42.50%; M 57.50%)	38.69% (F 57.55%; M 42.45%)	26.46% (F 65.52%; M 34.48%)	20.26% (F 73.87%; M 26.13%)	< 0.001
**Psychological constructs**							
Ineffectiveness Composite	54.51 ± 27.35 (F 62.35 ± 1.41; M 42.37 ± 1.72)	*p* < 0.001	16.42% (F 34.44%; M 65.56%)	44.89% (F 54.47%; M 45.53%)	21.53% (F 73.73%; M 26.27%)	17.15% (F 86.17%; M 13.83%)	< 0.001
Eating Disorder risk Composite	46.31 ± 29.02 (F 57.38 ± 1.52; M 29.26 ± 1.49)	*p* < 0.001	28.57% (F 33.33%; M 66.67%)	39.38% (F 55.35%; M 44.65%)	20.70% (F 89.39%; M 10.62%)	11.36% (F 95.16%; M 4.84%)	< 0.001
Interpersonal Problems Composite	64.29 ± 37.50 (F 62.89 ± 1.55; M 49.63 ± 1.84)	*p* < 0.001	16.27% (F 48.31%; M 51.69%)	38.21% (F 53.11%; M 46.89%)	22.85% (F 65.60%; M 34.40%)	22.67% (F 77.42%; M 22.58%)	< 0.001
Affective Problems Composite	64.29 ± 37.50 (F 71.93 ± 1.33; M 52.46 ± 3.37)	*p* < 0.001	10.58% (F 29.31%; M 70.69%)	39.05% (F 48.13%; M 51.87%)	21.72% (F 68.07%; M 31.93%)	28.65% (F 84.08%; M 15.92%)	< 0.001
Overcontrol Composite	61.70 ± 37.00 (F 65.74 ± 1.40; M 55.46 ± 3.35)	*p* < 0.001	11.31% (F 48.39%; M 51.61%)	39.42% (F 50.46%; M 49.54%)	29.01% (F 64.78%; M 35.22%)	20.26% (F 81.98%; M 18.02%)	< 0.001
Global Psychological Maladjustment	62.31 ± 24.68 (F 70.07 ± 1.24; M 50.55 ± 1.57)	*p* < 0.001	9.06% (F 30.61%; M 69.39%)	41.22% (F 47.98%; M 52.02%)	28.10% (F 63.82%; M 36.18%)	21.63% (F 91.45%; M 8.55%)	< 0.001

F = females; M = males

#### Questionnaire for the assessment of psychopathology in adolescence (Q-PAD)

In Fig. 1, Panel B, we display the percentages of individuals with one or more pathological Q-PAD scales. Table 3 presents the mean values, prevalence, and intensity for each Q-PAD scale. The Q-PAD findings suggest the presence of psychological distress leading to discomfort or challenging situations that may necessitate intervention in a percentage of adolescents, ranging from 2.93% for psychosocial risks to 23.77% for anxiety. The prevalence of body dissatisfaction, anxiety, depression, substance abuse, interpersonal conflicts, and family problems was higher in females compared to males (all *p* < 0.01). When considering different age groups (15-15.99 years, 16-16.99 years, 17-17.99 years, 18 years), no significant differences were noted in scale scores, except for substance abuse (*p* < 0.001) and self-esteem and well-being (*p* = 0.02), which were more frequently present with clear discomfort in the 16-16.99 years age group.

**Table 3:** Questionnaire for the assessment of psychopathology in adolescence (Q-PAD) scales

Scale	Mean value ±standard deviation	Score % of subjects (*n* = 548) (% of pathological subjects according to gender)				*p* females vs. males	
		0–84 = normal	85–90 = borderline psychological dystress	91 e 95 = presence of psychological dystress symptoms	> 95 = presence of psychological dystress symptoms that may require intervention		
Body dissatisfaction	60.19 ± 25.06 (F 63.22 ± 1.44; M 55.52 ± 1.52)	74.77% (F 56.23%; M 43.77%)	10.24% (F 62.58%; M 37.50%)	9.14% (F 90.0%; M 10.0%)	5.85% (F 68.75%; M 31.25%)	< 0.001	
Anxiety	66.97 ± 26.86 (F 72.66 ± 1.32; M 55.19 ± 1.93)	65.81% (F 54.17%; M 45.83%)	10.42% (F 54.39%; M 45.61%)	9.51% (F 78.85%; M 21.15%)	14.26% (F 83.33%; M 16.67%)	< 0.001	
Depression	62.64 ± 26.95 (F 66.64 ± 1.40; M 56.52 ± 1.90)	72.84% (F 54.66%; M 45.34%)	11.01% (F 83.33%; M 16.67%)	8.99% (F 71.43%; M 28.57%)	7.16% (F 71.79%; M 28.21%)	< 0.001	
Substance abuse	54.75 ± 27.31 (F 59.65 ± 1.44; M 47.24 ± 1.85)	82.57% (F 56.89%; M 43.11%)	8.08% (F 86.36%; M 13.64%)	5.32% (F 58.62%; M 41.38%)	4.04% (F 86.36%; M 13.61%)	< 0.001	
Interpersonal conflicts	66.34 ± 24.61 (F 69.90 ± 1.29; M 60.84 ± 1.71)	73.31% (F 56.61%; M 43.39%)	7.86% (F 65.12%; M 34.88%)	7.86% (F 76.74%; M 23.26%)	10.97% (F 73.33%; M 26.67%)	< 0.001	
Family problems	61.51 ± 24.96 (F 65.15 ± 1.33; M 55.89 ± 1.70)	78.79% (F 57.08%; M 42.92%)	10.79% (F 69.49%; M 30.51%)	3.84% (F 61.90%; M 38.10%)	6.58% (F 88.89%; M 11.11%)	0.001	
Uncertainty about the future	62.85 ± 27.05 (F 65.12 ± 1.46; M 59.34 ± 1.85)	73.13% (F 58.0%; M 42%)	5.48% (F 70.0%; M 30.0%)	8.59% (F 59.57%; M 40.43%)	12.80% (F 72.86%; M 27.14%)	0.01	
Psychosocial risks	33.58 ± 27.17 (F 39.5 ± 1.5; M 24.51 ± 1.66)	94.50% (F 59.42%; M 40.58%)	2.57% (F 78.57%; M 21.43%)	2.20% (F 83.33%; M 16.67%)	0.73% (F 75.0%; M 25.0%)	< 0.001	
**Scale**	**Mean value** **±standard deviation**	**0 e 20 = indicatori di autostima/benessere scarsi o nulli**,** con significative ripercussioni sullo stato psicologico compressivo della persona**	**21 e 40 = condizione psicologica di malessere**	**41 e 60 = discreto ma non ottimale stato di benessere**	**61 e 94 = stato di benessere nella norma**	**> 95 = segnalano una possibile esagerazione dello stato di autostima e benessere il cui significato va approfondito**	**p** **females vs. males**
Self-esteem and well-being	33.66 ± 25.31 (F 32.1 ± 1.55; M 36.04 ± 1.79)	39.89% (F 58.53%; M 41.47%)	30.88% (F 65.48%; M 34.52%)	12.58% (F 60.29%; M 39.71%)	14.52% (F 54.43%; M 45.57%)	2.21% (F 66.67%; M 33.33%)	0.07

F = females; M = males

#### Features of the population with psychopathological traits and symptomatology associated with EDs

Table 4 reports the percentages of patients with pathological EDI-3 specific and composite scales (≥ 67) associated with pathological general psychological scales (≥ 67) and pathological Q-PAD scales (≥ 91); sex differences are also described. Several significant differences in family features between populations with and without psychopathological traits and symptoms associated with eating disorders were recorded. Specifically:- Familiarity with eating disorders was observed in subjects with a pathological score for Drive for Thinness (*p* < 0.001, without a difference between sexes).

**Table 4:** Percentages of patients with EDI-3 pathological ED specific and compostite scales (≥67) associated with pathological general psychological scales (≥67) and pathological Q-PAD scales (≥91; for self-esteem and well-being scales ≤ 40)

	Drive for Thinness (≥67) *n* = 159		*p* F vs. M	Bulimia (≥67) *n* = 282	*p* F vs. M		Body Dissatisfaction (≥67) *n* = 147	*p* F vs. M	Eating Disorder Risk Composite (≥67) *n* = 150	*p* F vs. M
**ED specific and compostite scales (≥67) as % of subjects (% of pathological subjects according to gender)**										
.Low Self-Esteem	60.38% (F 94.79%; M 5.21%)		0.01	49.29% (F 83.45%; M 16.55%)	< 0.001		64.63% (F 93.68%; M 6.32%)	0.07	62.86% (F 92.73%; M 7.27%)	0.42
Personal Alienation	59.75% (F 93.68%; M 6.32%)		0.05	53.19% (F 82.0%; M 18.0%)	< 0.001		65.99% (F 92.78%; M 7.22%)	0.18	64.57% (F 92.04%; M 7.96%)	0.69
Interpersonal Insecurity	61.64% (F 92.86%; M 7.14%)		0.12	54.09% (F 80.92%; M 19.08%)	< 0.001		62.33% (F 94.51%; M 5.49%)	0.03	62.07% (F 92.59%; M 7.41%)	0.46
Interpersonal Alienation	51.57% (F 95.12%; M 4.88%)		0.02	43.62% (F 83.74%; M 16.26%)	< 0.001		50.34% (F 94.59%; M 5.41%)	0.08	53.14% (F 86.67%; M 13.33%)	0.10
Interoceptive Deficits	80.50% (F 92.97%; M 7.03%)		0.01	68.08% (F 82.81%; M 17.19%)	< 0.001		82.99% (F 92.62%; M 7.38%)	0.05	80.57% (F 93.62%; M 6.38%)	0.03
Emotional Dysregulation	71.07% (F 93.01%; M 6.19%)		0.01	64.89% (F 80.87%; M 19.13%)	< 0.001		74.15% (F 92.66%; M 7.34%)	0.12	73.14% (F 92.97%; M 7.03%)	0.23
Maturity fears	62.26% (F 91.92%; M 8.08%)		0.28	52.48% (F 79.05%; M 20.95%)	< 0.01		61.22% (F 92.22%; M 7.78%)	0.36	61.71% (F 92.59%; M 7.41%)	0.48
Perfectionism	61.01% (F 93.81%; M 6.19%)		0.04	53.55% (F 78.81%; M 21.19%)	< 0.01		61.90% (F 94.51%; M 5.49%)	0.02	60.57% (F 96.23%; M 3.77%)	< 0.01
Ascetism	76.73% (F 90.98%; M 9.02%)		0.42	63.48% (F 78.21%; M 21.79%)	< 0.01		81.63% (F 91.67%; M 8.33%)	0.30	77.71% (F 91.18%; M 8.82%)	0.82
**Q-PAD scales (≥91)**										
Body dissatisfaction	47.17% (F 86.67%; M 13.33%)	0.19		24.47% (F 85.51%; M 14.49%)	< 0.01	43.30% (F 90.14%; M 9.86%)		0.89	42.86% (F 88.0%; M 12.0%)	0.16
Anxiety	41.51% (F 95.45%; M 4.55%)	0.05		33.69% (F 85.26%; M 14.75%)	< 0.001	41.50% (F 91.80%; M 8.20%)		0.64	40.57% (F 94.37%; M 5.63%)	0.25
Depression	28.38% (F 91.11%; M 8.9%)	0.75		25.53% (F 75%; M 25%)	0.37	31.29% (F 89.13%; M 10.87%)		0.70	30.29% (F 88.68%; M 11.32%)	0.39
Substance abuse	12.58% (F 90.0%; M 10.0%)	0.99		15.30% (F 74.42%; M 25.58%)	0.57	13.61% (F 95.0%; M 5%)		0.45	13.71% (F 9167%; M 8.33%)	0.96
Interpersonal conflicts	38.82% (F 95.92%; M 4.08%)	0.09		27.66% (F 79.49%; M 20.51%)	0.05	31.97% (F 91.49%; M 8.51%)		0.77	32.0% (F 91.07%; M 8.93%)	0.90
Family problems	17.61% (F 96.43%; M 3.57%)	0.20		16.31% (F 86.96%; M 13.04%)	< 0.01	18.37% (F 96.38%; M 3.70%)		0.25	18.29% (F 96.88%; M 3.12%)	0.22
Uncertainty about the future	26.42% (F 90.48%; M 9.52%)	0.89		26.60% (F 76.0%; M 24.0%)	0.25	25.85% (F 92.11%; M 7.89%)		0.69	26.86% (F 91.49%; M 8.51%)	0.98
Psychosocial risks	3.8% (F 100%; M 0%)	0.48		4.27% (F 91.67%; M 8.33%)	0.10	5.44% (F 100%; M 0%)		0.34	4.6% (F 100%; M 0%)	0.37
**Q-PAD scales (**≤ 40**) as % of subjects (% of pathological subjects according to gender)**										
Self-esteem and well-being	35.32% (F 91.18%; M 8.82%)	0.17		55.58% (F 73.36%; M 26.64%)	0.09	34.81% (F 89.55%; M 10.45%)		0.23	39.69% (F 90.13%; M 9.87%)	0.12

F = females; M = males

- Low levels of parental education (*p* < 0.01, without a difference between sexes) and familiarity with eating disorders (*p* < 0.01, more prevalent in females, *p* < 0.001) were associated with a pathological score for Bulimia.
- Low levels of parental education (*p* < 0.01) and previous psychopharmacological treatments (*p* = 0.03, without a difference between sexes) were associated with a pathological score in Body Dissatisfaction.
- Familiarity with eating disorders (*p* < 0.001, without a difference between sexes) was noted in subjects with a pathological score for the Eating Disorder Risk Composite.

#### Correlation between EDI-3 and Q-PAD scales

We observed significant correlations between Q-PAD Body Dissatisfaction and EDI-3 Body Dissatisfaction (*r* = 0.7), Q-PAD Interpersonal Conflicts and EDI-3 Interpersonal Problems (*r* = 0.6), and Q-PAD Self-esteem and Well-being and EDI-3 Ineffectiveness Composite (*r*=-0.7). Additionally, moderate correlations were noted between Q-PAD Anxiety and EDI-3 Emotional Dysregulation (*r* = 0.48) and Q-PAD Depression and EDI-3 Emotional Dysregulation (*r* = 0.46).

## Discussion

Our study revealed a high prevalence of psychopathological traits and symptomatology associated with eating disorders (EDs) and psychological distress among Italian high school adolescents, particularly among females. We observed an association and correlation between EDs symptoms and psychological distress, highlighting the role of general psychological maladjustment in the development of EDs. Additionally, family attitudes and unhealthy lifestyle behaviors appeared to be partially correlated with EDs pathology and psychopathological profiles, respectively. Early detection of these often underestimated health problems can facilitate timely interventions and the implementation of preventive programs. Adolescence is a critical period characterized by high-risk behaviors [35, 36] and is often associated with psychological distress and other mental health issues [37–39].

Psychological distress encompasses non-specific mental health conditions characterized by anxiety, depression, and somatic symptoms [38]. It affects various aspects of adolescents’ daily lives, such as school performance, relationships with family and friends, and has been linked to an increased risk of EDs [40]. Our study focused on psychological distress and symptoms associated with EDs, including body dissatisfaction, low self-esteem, depression, anxiety, perfectionism, interoceptive deficits, ascetism, emotional dysregulation, maturity fears, interpersonal insecurity, and alienation, overcontrol, interpersonal and affective problems, global psychological maladjustment. Using a self-administered screening tool, we identified the presence of at least one trait or symptom associated with EDs, with clinical interest, in a percentage of individuals ranging from 2.93% for psychosocial risk to 51.83% for interoceptive deficits. Specifically, subjects displayed interoceptive deficits (51.83%), interpersonal insecurity (48.08%), emotional dysregulation (47.99%), and ascetism (46.72%). Furthermore, 70% of cases reported low levels of self-esteem and well-being, highlighting the existence of severe discomfort among high school Italian adolescents. Additionally, we observed a homogeneous distribution of pathological scores across all age ranges, indicating widespread psychological distress.

Our results confirm previous studies linking EDs and psychopathological disorders [2, 5, 13, 41–44], such as a study by Criscuolo et al., which investigated the association between EDs and psychopathology in 122 adolescents suffering from EDs [41]. The researchers found a high correlation between the two conditions [41]. Another study by Sander et al. examined the association between anxiety, depression, and ED-related impairment in 320 females aged 12 to 25 years [39]. The authors found that high levels of impairment in anxiety and depression were associated with more severe ED symptoms [39]. Swanson et al. also found high rates of comorbid psychiatric disorders and/or ‘impairment’ in ED patients, with psychological distress observed in about 97% of anorexia nervosa (AN) patients, 78% in bulimia nervosa (BN) patients, and 62.6% in binge-eating disorder (BED) patients [5]. Similar results were obtained by Giel et al., who investigated the prevalence of ED symptoms and their potential relationship with weight change, general psychopathology, and health-related quality of life in 41 obese adolescents [42]. They found that 43% of the patients screened positive for ED pathology, and this subgroup displayed a higher psychopathological burden compared to those who tested negative [42]. These findings underscore the close overlap and mutual interaction between EDs and psychological distress in children and adolescents, highlighting the need for an integrated approach to both prevention and treatment of these conditions.

The data from our study is particularly relevant because, as proposed by Prefit et al. in a recent meta-analysis, emotional distress, such as anxiety and depression, and EDs share a common etiology [19]. The authors suggest that disordered eating behaviors represent maladaptive strategies for regulating emotional states [19]. This suggests that these behaviors may lead to greater eating disorder psychopathology, and vice versa [2, 20]. This opens up new treatment approaches that consider EDs not as isolated conditions but as part of a broader psychological distress.

As proposed by Fairburn et al., the transdiagnostic theory of EDs considers low self-esteem, mood intolerance, perfectionism, and interpersonal difficulties as key maintaining factors [21]. In particular, they suggest that some patients exhibit additional maintaining processes that interact with the core eating disorder mechanisms. Among these additional processes are perfectionism, pervasive low self-esteem, intense mood intolerance, and interpersonal difficulties [21]. Elevated levels of perfectionism are also found in anxiety disorders and depression [2]. Thus, for these patients, addressing clinical perfectionism may be an effective strategy for managing comorbid EDs.

Furthermore, adverse mood states can influence eating habits and exacerbate EDs. It is well-known that adverse mood states can trigger binge eating, with their primary effect being to disrupt dietary restraint [21]. Rather than accepting changes in mood and handling them appropriately, some patients, especially adolescents, engage in what is called “dysfunctional mood modulatory behavior.” This behavior may manifest as binge eating, self-induced vomiting, and/or intense exercise, with binge eating being the most common. For these patients, such behaviors become habitual means of regulating mood [21].

Low self-esteem is a common issue among teenagers and may be associated with depression and anxiety [45]. Body dissatisfaction (found in 26.82% of our sample) contributes to unhealthy eating behaviors and possible restrictions [46]. Interestingly, Brockmeyer et al. highlighted that individuals with low self-esteem may attempt to boost their self-esteem by controlling food, weight, or body shape, and achieving a low weight (strongly associated with excessive eating control) can be perceived as a victory [45, 47]. Furthermore, disordered weight control behaviors and symptoms do not necessarily meet psychiatric criteria for an ED diagnosis [48]; they are estimated to be 20 times more common in the general population [49] compared to those behaviors and symptoms that meet diagnostic criteria. Therefore, it is crucial to address psychological aspects when dealing with EDs to address both the physical and psychological consequences of the disorder.

Our findings confirmed a female predominance in symptomatology associated with EDs [2]. This can be explained by the additional factors that may increase the risk of EDs in females, such as social pressure, which is often expressed through media messages promoting the ideal of beauty and thinness. In males, emotional distress is likely the primary factor that preoccupies them with eating behaviors [40].

Familiar attitudes seem to be partially correlated with EDs pathology. We found a significant correlation between familiarity with EDs and pathological scores for the drive for thinness and bulimia. Additionally, low levels of parental education were found in subjects with pathological scores for bulimia and higher levels of body dissatisfaction. These findings are supported by current literature [50, 51]. A systematic review by Marcos et al. used meta-analysis procedures to assess the relationships between EDs and family influence [50]. The authors highlighted the strong influence of the family on dieting behavior, body dissatisfaction, and bulimic symptoms in adolescent girls and boys [50].

In our population, a small percentage of individuals were underweight (1.1%) and overweight/obese (9.1%). Inadequate weight control may be related to the reported unhealthy lifestyle behaviors observed in both females and males. Furthermore, it is important to consider that EDs involve an unhealthy relationship with food [31, 35] and disordered eating behaviors cannot be ruled out, especially in the presence of psychological distress. These findings align with theories that suggest individuals with ED symptoms may also experience comorbid psychological distress, such as interoceptive deficits, emotional dysregulation, ascetism, body dissatisfaction, and anxiety.

Regarding our sample, it is possible to outline the clinical and psychopathological profile of adolescents at risk of developing an eating disorder. These individuals have poor interoceptive abilities, struggle to identify and name their emotional experiences, and are overwhelmed by the intensity of their emotions, leading to anxiety (as indicated by the Anxiety Scale) [52]. Their difficulty in recognizing their feelings hinders their ability to regulate negative emotions and affective states through functional strategies (as seen in the Emotional Dysregulation scale). In a social context where external image holds significant value, these adolescents attach great importance to their bodies. In our sample, the body is often perceived as inadequate and unsatisfactory (as indicated by the Body Dissatisfaction scale), leading to a more general sense of inadequacy (Self-Esteem and Well-being) [53–55]. Interestingly, during a period of rapid transformation, such as adolescence, rigid control of bodily needs gives adolescents an illusory sense of strength and self-worth (ascetism). Controlling their bodies through inappropriate eating behaviors becomes a way to manage emotions that would otherwise be difficult to tolerate [56]. Emotional dysregulation, widely observed in our sample, is also one of the underlying mechanisms in the frequent clinical observation that a restrictive eating disorder often leads to dysregulated eating behaviors with or without compensatory behaviors [57].

Moreover, EDs often present a continuum, ranging from restriction to binge eating, and over time, an anorexic individual can transition to bulimia, or vice versa. This aligns with the results of our study, where interoceptive deficits, ascetism, perfectionism, dissatisfaction with the body, and emotional dysregulation are characteristics present in a high percentage of both the drive for thinness and bulimia, supporting the notion that EDs, particularly in adolescence, represent a broader disorder that is not always so rigidly classifiable.

Shortening the time between the onset of symptoms and the start of treatment improves the prognosis for EDs. Early identification of symptoms can alter the trajectory of EDs and their chronic health consequences [58, 59]. Understanding the extent of psychological distress that may coexist with ED psychological symptoms is crucial for devising effective intervention strategies.

Our study has certain limitations that should be taken into account when interpreting the results. We decided to report and analyze the results based on two main demographic features: gender and age. Specifically, we focused on gender because epidemiological data suggest that EDs are more commonly diagnosed in females.

However, the results obtained have shown a situation worse than anticipated. Therefore, exploring other predictive variables and describing the results based on these variables will be very useful in better understanding the phenomenon. This paper can be considered as a basis (“generative of hypothesis”) for further works designed ad hoc that will investigate relationships between EDs and distress.

As an additional limitation, we used a self-reported instrument to assess symptomatology associated with EDs and psychopathology, without diagnostic interviews, which could introduce reporting bias; other self-report biases may have occurred, as some questions required recalling past history, making them susceptible to recall bias. Furthermore, no data on non-pharmacological treatments (e.g. psychotherapy) and the presence of other clinical conditions have been recorded. Longitudinal research is needed to confirm EDs diagnoses and gain a more comprehensive understanding of the potential relationships between EDs and psychological distress. Finally, it could be interesting to include subjects from other Italian regions where the social context may differ significantly, potentially affecting the reported symptoms. This could lead to a better understanding of the predictor variables.

## Conclusions

Symptoms of EDs and psychological distress may represent an underestimated issue in adolescents. These disorders can coexist, creating a vicious circle that delays correct diagnosis and worsens the prognosis. Early detection and intervention have the potential to improve treatment outcomes. Population screening in high schools may be a valuable strategy for identifying young individuals in need of clinical evaluation for EDs and psychological distress.

### Electronic supplementary material

Below is the link to the electronic supplementary material.

Supplementary Material 1

## Data Availability

All data are reported in the paper.

## Abbreviations

BMI: Body mass index
EDs: Eating disorders
EDI: 3-Eating Disorder Inventory-3
Q-PAD: Questionnaire for the Assessment of Psychopathology in Adolescence
SD: Standard deviation
CI: Confidence interval
AN: Anorexia nervosa
BN: Bulimia nervosa
BED: Binge-eating disorder
