# Supplementary material for: Eating disorder risks and psychopathological distress in Italian high school adolescents
Source: Ital J Pediatr. 2024 Aug 7;50:144. doi: 10.1186/s13052-024-01717-7 (PMC11304653; doi:10.1186/s13052-024-01717-7)
Supplement: Supplementary file 1 — Supplementary Material 1 [file 13052_2024_1717_MOESM1_ESM.pdf]

OSPEDALE DEI BAMBINI
Vittore Buzzi

Sistema Socio Sanitario
Regione Lombardia
ASST Fatebenefratelli Sacco

UNIVERSITÀ DEGLI STUDI DI MILANO

SCHEDA STUDENTE **FEMMINA**

Titolo del Progetto "Diamo un peso al disagio giovanile": screening e prevenzione del disagio psicologico dietro ai disturbi alimentari in età adolescenziale

ID studente:______________________________

Sesso ☐M ☐F

Data di nascita: _________

Da quante persone è composta la tua famiglia, te compreso? ____________

Indica il nome della scuola e la classe che frequenti

Quante ore dormi?
☐ <6
☐ <8
☐ >8

Fai la prima colazione ?
□ sempre
□ spesso
□ raramente
□ mai

Quale bevanda consumi a colazione ?
□ latte/yogurt/caffèlatte/cappuccino
□ succo di frutta/spremuta
□ tè/caffè
□ cioccolata

La tua colazione è a base principalmente di:
□biscotti/fettebiscottate/merendine/ cereali da prima colazione/pane
□ frutta
□ salumi e formaggi
□ pizza/focaccia/toast

Consumi almeno due porzioni di frutta al giorno ?
□ sempre
□ spesso
□ raramente
□ mai

Consumi almeno due porzioni di verdura al giorno ?
□ sempre
□ spesso
□ raramente
□ mai

I tuoi pasti si concludono abitualmente con un dessert o un dolce ?
□ sempre
□ spesso
□ raramente
□ mai

Ai tuoi pasti, oltre all'acqua, bevi abitualmente vino o birra ?
□ sempre
□ spesso
□ raramente
□ mai

Consumi abitualmente tutti i giorni colazione, pranzo e cena ?
- □ sempre
- □ spesso
- □ raramente
- □ mai

Le bevande che consumi più spesso fuori pasto sono :
- □ acqua
- □ bibite zuccherine (cola, aranciata, tè freddo, gassosa, acqua tonica, ecc.)
- □ birra/vino
- □ succhi di frutta/spremute/frullati

Consumi almeno un bicchiere di latte o un vasetto di yogurt al giorno?
- □ sempre
- □ spesso
- □ raramente
- □ mai

Consumi almeno 1-1,5 litri di acqua al giorno?
- □ sempre
- □ spesso
- □ raramente
- □ mai

Svolgi regolarmente qualche attività fisica ?
- □ sempre, tutto l'anno
- □ solo in alcune stagioni
- □ talvolta
- □ mai

Quanto tempo dedichi all'attività fisica (sci fondo, sci discesa, palestra, nuoto, basket, calcio, danza, ecc.) ?
- □ 1h-2h alla settimana
- □ 3h-4h alla settimana
- □ più di 4h alla settimana
- □ non svolgo nessuna attività fisica

Quando hai del tempo libero, cosa preferisci fare ?
- □ una passeggiata
- □ guardare la TV/ ascoltare musica/ giocare con il computer/leggere
- □ praticare sport
- □ fare shopping

Quanto tempo trascorri davanti al computer e/o televisore ?
- □ 1h-2h al giorno
- □ 3h-4h al giorno
- □ 5h-6h al giorno
- □ più di 6h al giorno

Le ore di educazione fisica che pratichi a scuola:
- □ ti stancano fisicamente
- □ ti annoiano
- □ ti stimolano a praticare sport anche al di fuori della scuola
- □ ti "fanno sentire meglio"

Il tuo stile di vita è:
- □ molto sedentario
- □ sedentario
- □ moderatamente attivo
- □ molto attivo

Claudio Sica, Luigi Rocco Chiri,
Riccardo Favilli e Igor Marchetti

Q-PAD

FASCICOLO PER IL SOGGETTO (FEMMINE)

ISTRUZIONI

Di seguito troverai 81 affermazioni relative ad atteggiamenti e comportamenti più o meno comuni. Ti preghiamo di valutare in che misura ciascuna affermazione descrive te stessa o le tue opinioni, utilizzando lo schema di risposta seguente:

1 = l'affermazione è falsa/non descrive per nulla la mia situazione o opinione

2 = l'affermazione è parzialmente falsa/descrive poco la mia situazione o opinione

3 = l'affermazione è abbastanza vera/descrive abbastanza bene la mia situazione o opinione

4 = l'affermazione è vera/descrive accuratamente la mia situazione o opinione

Ricorda che non ci sono risposte giuste o sbagliate. Cerca di rispondere in base a quanto l'affermazione ti descrive correttamente, non in base a quello che pensi dovrebbe essere la risposta giusta o a quello che gli altri risponderebbero. Procedi con attenzione, ma ricorda che spesso la prima risposta è la più accurata!

Per favore rispondi a tutte le affermazioni.

Buon lavoro!

Erickson

1	Sono soddisfatta del mio aspetto fisico	1	2	3	4
2	Mi sento spesso tesa	1	2	3	4
3	Molte persone mi irritano	1	2	3	4
4	Sono un'assidua frequentatrice di feste, party e discoteche	1	2	3	4
5	Sono soddisfatta di me stessa	1	2	3	4
6	Evito di parlare con i miei genitori	1	2	3	4
7	Penso che i miei fianchi siano troppo grandi	1	2	3	4
8	Quando sono agitata, ho difficoltà a respirare	1	2	3	4
9	Mi sembra di essere diversa dalla maggior parte dei miei coetanei	1	2	3	4
10	Mi affatica eseguire anche il più piccolo compito	1	2	3	4
11	Mi sembra di non trovare un'area di studio che si adatti a me	1	2	3	4
12	Spendo tanti soldi in alcol o altre sostanze	1	2	3	4
13	Sento che la mia vita sta trascorrendo bene come quella di molti altri della mia età	1	2	3	4
14	La mia famiglia non mi capisce	1	2	3	4
15	Sono quasi sempre preoccupata per qualcosa	1	2	3	4
16	Ultimamente, mi sento triste o malinconica per la maggior parte del tempo	1	2	3	4
17	Ho bisogno di conoscermi meglio per scegliere una professione	1	2	3	4
18	Mi è capitato di saltare i miei impegni scolastici o lavorativi perché sono stata fuori fino a tardi la sera precedente	1	2	3	4
19	Mi fido del mio giudizio	1	2	3	4
20	Il mio ambiente familiare a volte è davvero imprevedibile	1	2	3	4
21	Spesso mi verrebbe da prendere a pugni qualcuno	1	2	3	4
22	Penso che le mie cosce siano troppo grosse	1	2	3	4
23	In genere non vado molto d'accordo con le altre persone	1	2	3	4
24	Ho perso interesse nelle cose che mi hanno sempre divertita	1	2	3	4
25	Sono preoccupata perché non riesco a trovare una professione che mi interessi	1	2	3	4
26	Quando bevo a volte faccio cose che mi imbarazzano un po'	1	2	3	4
27	La mia famiglia cerca di gestire la mia vita	1	2	3	4

© 2011, C. Sica et al., *Q-PAD,* Trento, Erickson

28	Ho il terrore di aumentare di peso	1	2	3	4
29	Ultimamente ho avuto difficoltà a concentrarmi	1	2	3	4
30	Rimango delusa tutte le volte che do confidenza agli altri	1	2	3	4
31	Sono insoddisfatta perché non ho ancora progetti professionali per il futuro	1	2	3	4
32	Mi sono messa nei guai a causa del bere	1	2	3	4
33	Ultimamente sono spesso agitata	1	2	3	4
34	Le persone intorno a me non capiscono come sono veramente	1	2	3	4
35	Le cose sono andate di male in peggio	1	2	3	4
36	Sono preoccupata di non riuscire a trovare un corso di studi adatto a me	1	2	3	4
37	A volte usare alcol o altre sostanze mi aiuta a superare i miei problemi	1	2	3	4
38	I miei genitori non vogliono lasciarmi crescere	1	2	3	4
39	Se aumento di mezzo chilo sono preoccupata di continuare ad aumentare	1	2	3	4
40	Spesso sono così nervosa che sento il mio cuore martellare	1	2	3	4
41	Sono spesso coinvolta in discussioni	1	2	3	4
42	Ultimamente il tempo non mi passa mai	1	2	3	4
43	Non so con quale criterio scegliere una professione	1	2	3	4
44	Non ho nessuna particolare potenzialità o talento	1	2	3	4
45	Mi sento soffocata dai miei genitori	1	2	3	4
46	Mi sento in forma	1	2	3	4
47	Mi preoccupo di cose di cui non si preoccupa la maggior parte delle persone	1	2	3	4
48	Pensieri tristi mi tengono sveglia la notte	1	2	3	4
49	Sebbene sappia che è arrivato il tempo per me di decidere, non sono ancora pronta per scegliere un'area di studio o una professione	1	2	3	4
50	Alcuni mi fanno notare il mio uso di alcol o di altre sostanze	1	2	3	4
51	Non mi sento capace come la maggior parte delle persone	1	2	3	4
52	La mia vita familiare è piacevole e soddisfacente	1	2	3	4
53	Ho fiducia in me stessa	1	2	3	4
54	Do troppa importanza al peso	1	2	3	4

© 2011, C. Sica et al., *Q-PAD*, Trento, Erickson

55	Penso di mostrare i segni di molto stress	1	2	3	4
56	Non vado d'accordo con i professori e le altre autorità	1	2	3	4
57	Dalle mie attività non ottengo lo stesso piacere di un tempo	1	2	3	4
58	Sento di essere forzata in una professione che non voglio	1	2	3	4
59	Le persone si sono approfittate di me mentre ero ubriaca o alticcia	1	2	3	4
60	Molto spesso agisco in modo da dovermi poi pentire	1	2	3	4
61	Mi godo davvero la vita	1	2	3	4
62	Sono assillata dal desiderio di essere più magra	1	2	3	4
63	Ultimamente le mie preoccupazioni hanno reso difficile addormentarmi	1	2	3	4
64	Sono stanca del modo in cui le persone mi trattano	1	2	3	4
65	Non mi importa quello che faccio, tanto le cose non miglioreranno	1	2	3	4
66	Sono ansiosa perché mi è rimasto poco tempo per cercare una professione	1	2	3	4
67	Ho un'opinione molto positiva di me stessa	1	2	3	4
68	Non mi piace stare a casa perché nella mia famiglia si discute sempre	1	2	3	4
69	Mi sento spesso inquieta ma non so il perché	1	2	3	4
70	Sbaglio spesso nella scelta delle mie amicizie	1	2	3	4
71	Mi sembra di non potermi liberare dalla mia tristezza	1	2	3	4
72	I miei amici hanno un'idea migliore del loro futuro rispetto a quella che io ho del mio	1	2	3	4
73	Qualcuno dei miei amici non approva l'utilizzo che faccio dell'alcol o di altre sostanze	1	2	3	4
74	Le persone dicono che manco di fiducia in me stessa	1	2	3	4
75	Sono infastidita da pensieri da cui non riesco a liberarmi	1	2	3	4
76	Non ho fiducia nella maggior parte delle persone intorno a me	1	2	3	4
77	Sono molto indecisa sul mio futuro	1	2	3	4
78	A volte bere o usare altre sostanze mi mette nei guai o in situazioni pericolose	1	2	3	4
79	Mi sento frequentemente insoddisfatta della persona che sono	1	2	3	4
80	Ho paura dei conflitti con i miei genitori	1	2	3	4
81	Ho molta resistenza alla fatica	1	2	3	4

© 2011, C. Sica et al., *Q-PAD*, Trento, Erickson

EDI-3
Eating Disorder Inventory-3

David M. Garner
Adattamento italiano a cura di Marco Giannini, Linda Pannocchia, Riccardo Dalle Grave, Filippo Muratori e Valentina Viglione

Rispondi a tutte le domande contenute in questa pagina. Dopodiché, volta pagina e segui attentamente le istruzioni.

A. Peso attuale kg _____

B. Altezza cm _____

C. Qual è il peso più alto che hai mai raggiunto (escluse eventuali gravidanze)? Kg _____
Quanto tempo fa hai raggiunto questo peso per la prima volta? _____
Per quanto tempo hai mantenuto questo peso? _____

D. Qual è il peso più basso che hai raggiunto da adulto (o nell'adolescenza prima di 18 anni)? Kg _____
Quanto tempo fa hai raggiunto questo peso per la prima volta? _____ mesi fa
Per quanto tempo hai mantenuto questo peso _____ mesi

E. Quale peso hai mantenuto per il periodo di tempo più lungo? Kg _____
A che età hai raggiunto questo peso per la prima volta? _____ anni

F. Se il tuo peso è cambiato molto negli anni, c'è un peso al quale cerchi di ritornare quando non sei a dieta? □ Sì □ No
Se sì, qual è questo peso? Kg _____
A quale età hai raggiunto questo peso per la prima volta? _____ anni

G. Qual è il maggior numero di chili che hai mai perso? Kg _____
Li hai persi di proposito? □ Sì □ No
Che peso hai raggiunto dimagrendo? Kg _____
A che età hai raggiunto questo peso? _____ anni

H. Secondo te, quale sarebbe il tuo peso se non cercassi consapevolmente di controllarlo? Kg _____

I. Quanto ti piacerebbe pesare? Kg _____

J. A che età sono iniziati i problemi di peso (se ve ne sono)? _____ anni

K. Professione del padre ____

L. Professione della madre ____

Istruzioni

Le domande che seguono riguardano atteggiamenti, sentimenti e comportamenti relativi alla tua alimentazione e a te stesso/a.

Per ciascuna domanda decidi quale risposta corrisponde meglio a te scegliendo tra **A = SEMPRE, B = DI SOLITO, C = SPESSO, D = TALVOLTA, E = RARAMENTE, F = MAI**, e segnala con una crocetta. Ad esempio, se scegli la risposta SPESSO metti la crocetta sulla lettera C.

Rispondi a tutte le domande senza tralasciarne alcuna. **NON CANCELLARE!** Se vuoi cambiare la risposta, fai un cerchio intorno alla lettera sbagliata e metti la crocetta sulla lettera giusta.

A= SEMPRE; B= DI SOLITO; C=SPESSO; D= TALVOLTA; E= RARAMENTE; F=MAI

1.	Mangio dolci e carboidrati senza sentirmi nervoso/a	A	B	C	D	E	F
2.	Penso che il mio stomaco sia troppo grande	A	B	C	D	E	F
3.	Vorrei poter ritornare alla sicurezza dell'infanzia	A	B	C	D	E	F
4.	Mangio quando sono di cattivo umore	A	B	C	D	E	F
5.	Mi rimpinzo di cibo	A	B	C	D	E	F
6.	Vorrei essere più giovane	A	B	C	D	E	F
7.	Ho il pensiero della dieta	A	B	C	D	E	F
8.	Mi spavento quando i miei sentimenti sono troppo forti	A	B	C	D	E	F
9.	Penso che le mie cosce siano troppo grosse	A	B	C	D	E	F
10.	Mi sento una persona incapace	A	B	C	D	E	F
11.	Mi sento estremamente in colpa dopo avere mangiato troppo	A	B	C	D	E	F
12.	Penso che la grandezza del mio stomaco sia proprio giusta	A	B	C	D	E	F
13.	Nella mia famiglia sono accettati soltanto risultati eccellenti	A	B	C	D	E	F
14.	Il periodo più felice della vita è quando si è bambini	A	B	C	D	E	F
15.	Parlo dei miei sentimenti	A	B	C	D	E	F
16.	Ho il terrore di aumentare di peso	A	B	C	D	E	F
17.	Ho fiducia negli altri	A	B	C	D	E	F

18.	Mi sento solo/a al mondo	A	B	C	D	E	F
19.	Sono soddisfatto/a del mio aspetto fisico	A	B	C	D	E	F
20.	Generalmente sento le cose della mia vita sotto controllo	A	B	C	D	E	F
21.	Non riesco a capire chiaramente quale emozione sto provando	A	B	C	D	E	F
22.	Preferirei essere una persona adulta piuttosto che un/una bambino/a	A	B	C	D	E	F
23.	Riesco facilmente a comunicare con gli altri	A	B	C	D	E	F
24.	Vorrei essere qualcun altro	A	B	C	D	E	F
25.	Do troppa importanza al peso	A	B	C	D	E	F
26.	Riesco ad identificare chiaramente quale emozione sto provando	A	B	C	D	E	F
27.	Mi sento inadeguato/a	A	B	C	D	E	F
28.	Ho continuato ad abbuffarmi sentendo di non riuscire a fermarmi	A	B	C	D	E	F
29.	Da bambino/a facevo il possibile per non deludere i miei genitori e i miei insegnanti	A	B	C	D	E	F
30.	Ho rapporti stretti con gli altri	A	B	C	D	E	F
31.	Mi piace la forma del mio sedere	A	B	C	D	E	F
32.	Sono assillato/a dal desiderio di essere più magro/a	A	B	C	D	E	F
33.	Non so che cosa sta succedendo dentro di me	A	B	C	D	E	F
34.	Faccio fatica ad esprimere agli altri ciò che provo	A	B	C	D	E	F
35.	Il peso della vita adulta è troppo gravoso	A	B	C	D	E	F
36.	Non sopporto di non essere il/la migliore	A	B	C	D	E	F
37.	Mi sento sicuro/a di me stesso/a	A	B	C	D	E	F
38.	Ho il pensiero delle abbuffate	A	B	C	D	E	F
39.	Sono contento/a di non essere più un/una bambino/a	A	B	C	D	E	F

40.	Non riesco a capire se ho fame oppure no	A	B	C	D	E	F
41.	Ho una bassa stima di me stesso/a	A	B	C	D	E	F
42.	Sento di poter raggiungere le mete che mi prefiggo	A	B	C	D	E	F
43.	I miei genitori si aspettavano da me la perfezione	A	B	C	D	E	F
44.	Ho paura di perdere il controllo delle mie emozioni	A	B	C	D	E	F
45.	Penso che i miei fianchi siano troppo grandi	A	B	C	D	E	F
46.	Mangio moderatamente di fronte agli altri e mi rimpinzo quando sono andati via	A	B	C	D	E	F
47.	Mi sento gonfio/a dopo un pasto normale	A	B	C	D	E	F
48.	Penso che il periodo più felice sia quando si è bambini	A	B	C	D	E	F
49.	Se aumento di mezzo chilo sono preoccupato/a di continuare ad aumentare	A	B	C	D	E	F
50.	Sento di essere una persona che vale	A	B	C	D	E	F
51.	Quando sono di cattivo umore non so se sono triste, spaventato/a o arrabbiato/a	A	B	C	D	E	F
52.	Sento che devo fare le cose in maniera perfetta oppure non farle affatto	A	B	C	D	E	F
53.	Mi viene in mente di provare a vomitare per perdere peso	A	B	C	D	E	F
54.	Ho bisogno di tenere le persone ad una certa distanza (mi sento a disagio se qualcuno prova ad avvicinarsi troppo)	A	B	C	D	E	F
55.	Penso che la grandezza delle mie cosce sia giusta	A	B	C	D	E	F
56.	Mi sento vuoto/a emotivamente	A	B	C	D	E	F
57.	Riesco a parlare di ciò che penso e provo	A	B	C	D	E	F
58.	Il periodo migliore della vita è quando si diventa adulti	A	B	C	D	E	F
59.	Penso che il mio sedere sia troppo grande	A	B	C	D	E	F
60.	Provo sensazioni che non riesco bene a definire	A	B	C	D	E	F
61.	Mangio o bevo di nascosto	A	B	C	D	E	F

62.	Penso che i miei fianchi siano proprio della grandezza giusta	A	B	C	D	E	F
63.	Mi pongo obiettivi estremamente elevati	A	B	C	D	E	F
64.	Quando sono agitato/a o di cattivo umore ho paura di iniziare a mangiare	A	B	C	D	E	F
65.	Le persone a cui tengo veramente finiscono col deludermi	A	B	C	D	E	F
66.	Mi vergogno delle mie debolezze	A	B	C	D	E	F
67.	Gli altri potrebbero dire che sono emotivamente instabile	A	B	C	D	E	F
68.	Mi piacerebbe controllare completamente i bisogni del mio corpo	A	B	C	D	E	F
69.	Mi sento rilassato/a nella maggior parte delle situazioni di gruppo	A	B	C	D	E	F
70.	Dico impulsivamente cose che poi mi pento di avere detto	A	B	C	D	E	F
71.	Faccio di tutto per provare piacere	A	B	C	D	E	F
72.	Devo fare attenzione alla mia tendenza ad abusare di droghe	A	B	C	D	E	F
73.	Sono socievole con la maggior parte delle persone	A	B	C	D	E	F
74.	Mi sento intrappolato/a nei rapporti con gli altri	A	B	C	D	E	F
75.	L’astinenza mi fa sentire più forte spiritualmente	A	B	C	D	E	F
76.	Gli altri capiscono i miei veri problemi	A	B	C	D	E	F
77.	Non riesco a tenere lontano dalla mia mente pensieri strani	A	B	C	D	E	F
78.	Mangiare per il piacere è un segno di debolezza morale	A	B	C	D	E	F
79.	Sono incline a scatti di ira	A	B	C	D	E	F
80.	Credo che gli altri mi diano il credito che merito	A	B	C	D	E	F
81.	Devo stare attento/a alla mia tendenza ad abusare di alcolici	A	B	C	D	E	F
82.	Credo che rilassarsi sia semplicemente una perdita di tempo	A	B	C	D	E	F

83.	Gli altri potrebbero dire che mi irrito facilmente	A	B	C	D	E	F
84.	Mi sento perdente in ogni circostanza	A	B	C	D	E	F
85.	Ho forti sbalzi di umore	A	B	C	D	E	F
86.	Mi vergogno dei bisogni del mio corpo	A	B	C	D	E	F
87.	Preferisco trascorrere il tempo da solo/a piuttosto che con gli altri	A	B	C	D	E	F
88.	Le sofferenze rendono migliori	A	B	C	D	E	F
89.	So che la gente mi vuole bene	A	B	C	D	E	F
90.	Mi sento come se dovessi fare del male a me stesso/a o agli altri	A	B	C	D	E	F
91.	Penso di sapere realmente chi sono	A	B	C	D	E	F

SCHEDA STUDENTE

Titolo del Progetto "Diamo un peso al disagio giovanile": screening e prevenzione del disagio psicologico dietro ai disturbi alimentari in età adolescenziale

ID studente:______________________________

Sesso ☐M ☐F

Data di nascita: _________

Da quante persone è composta la tua famiglia, te compreso? ____________

Indica il nome della scuola e la classe che frequenti

Quante ore dormi?
- ☐ <6
- ☐ <8
- ☐ >8

Fai la prima colazione ?	□ sempre<br>□ spesso<br>□ raramente<br>□ mai
Quale bevanda consumi a colazione ?	□ latte/yogurt/caffèlatte/cappuccino<br>□ succo di frutta/spremuta<br>□ tè/caffè<br>□ cioccolata
La tua colazione è a base principalmente di:	□biscotti/fettebiscottate/merendine/ cereali da prima colazione/pane<br>□ frutta<br>□ salumi e formaggi<br>□ pizza/focaccia/toast
Consumi almeno due porzioni di frutta al giorno ?	□ sempre<br>□ spesso<br>□ raramente<br>□ mai
Consumi almeno due porzioni di verdura al giorno ?	□ sempre<br>□ spesso<br>□ raramente<br>□ mai
I tuoi pasti si concludono abitualmente con un dessert o un dolce ?	□ sempre<br>□ spesso<br>□ raramente<br>□ mai
Ai tuoi pasti, oltre all'acqua, bevi abitualmente vino o birra ?	□ sempre<br>□ spesso<br>□ raramente<br>□ mai

Consumi abitualmente tutti i giorni colazione, pranzo e cena ?
- □ sempre
- □ spesso
- □ raramente
- □ mai

Le bevande che consumi più spesso fuori pasto sono :
- □ acqua
- □ bibite zuccherine (cola, aranciata, tè freddo, gassosa, acqua tonica, ecc.)
- □ birra/vino
- □ succhi di frutta/spremute/frullati

Consumi almeno un bicchiere di latte o un vasetto di yogurt al giorno?
- □ sempre
- □ spesso
- □ raramente
- □ mai

Consumi almeno 1-1,5 litri di acqua al giorno?
- □ sempre
- □ spesso
- □ raramente
- □ mai

Svolgi regolarmente qualche attività fisica ?
- □ sempre, tutto l'anno
- □ solo in alcune stagioni
- □ talvolta
- □ mai

Quanto tempo dedichi all'attività fisica (sci fondo, sci discesa, palestra, nuoto, basket, calcio, danza, ecc.) ?
- □ 1h-2h alla settimana
- □ 3h-4h alla settimana
- □ più di 4h alla settimana
- □ non svolgo nessuna attività fisica

Quando hai del tempo libero, cosa preferisci fare ?
- □ una passeggiata
- □ guardare la TV/ ascoltare musica/ giocare con il computer/leggere
- □ praticare sport
- □ fare shopping

Quanto tempo trascorri davanti al computer e/o televisore ?
- □ 1h-2h al giorno
- □ 3h-4h al giorno
- □ 5h-6h al giorno
- □ più di 6h al giorno

Le ore di educazione fisica che pratichi a scuola:
- □ ti stancano fisicamente
- □ ti annoiano
- □ ti stimolano a praticare sport anche al di fuori della scuola
- □ ti "fanno sentire meglio"

Il tuo stile di vita è:
- □ molto sedentario
- □ sedentario
- □ moderatamente attivo
- □ molto attivo

Claudio Sica, Luigi Rocco Chiri,
Riccardo Favilli e Igor Marchetti

Q-PAD

FASCICOLO PER IL SOGGETTO (MASCHI)

Nome ____________	Cognome ____________	Classe ____________
Sesso ________	Età ________	Peso ________	Altezza ________

ISTRUZIONI

Di seguito troverai 81 affermazioni relative ad atteggiamenti e comportamenti più o meno comuni. Ti preghiamo di valutare in che misura ciascuna affermazione descrive te stesso o le tue opinioni, utilizzando lo schema di risposta seguente:

1 = l'affermazione è falsa/non descrive per nulla la mia situazione o opinione
2 = l'affermazione è parzialmente falsa/descrive poco la mia situazione o opinione
3 = l'affermazione è abbastanza vera/descrive abbastanza bene la mia situazione o opinione
4 = l'affermazione è vera/descrive accuratamente la mia situazione o opinione

Ricorda che non ci sono risposte giuste o sbagliate. Cerca di rispondere in base a quanto l'affermazione ti descrive correttamente, non in base a quello che pensi dovrebbe essere la risposta giusta o a quello che gli altri risponderebbero. Procedi con attenzione, ma ricorda che spesso la prima risposta è la più accurata!

Per favore rispondi a tutte le affermazioni.

Buon lavoro!

Erickson

1	Sono soddisfatto del mio aspetto fisico	1	2	3	4
2	Mi sento spesso teso	1	2	3	4
3	Molte persone mi irritano	1	2	3	4
4	Sono un assiduo frequentatore di feste, party e discoteche	1	2	3	4
5	Sono soddisfatto di me stesso	1	2	3	4
6	Evito di parlare con i miei genitori	1	2	3	4
7	Penso di essere troppo grasso	1	2	3	4
8	Quando sono agitato, ho difficoltà a respirare	1	2	3	4
9	Mi sembra di essere diverso dalla maggior parte dei miei coetanei	1	2	3	4
10	Mi affatica eseguire anche il più piccolo compito	1	2	3	4
11	Mi sembra di non trovare un'area di studio che si adatti a me	1	2	3	4
12	Spendo tanti soldi in alcol o altre sostanze	1	2	3	4
13	Sento che la mia vita sta trascorrendo bene come quella di molti altri della mia età	1	2	3	4
14	La mia famiglia non mi capisce	1	2	3	4
15	Sono quasi sempre preoccupato per qualcosa	1	2	3	4
16	Ultimamente, mi sento triste o malinconico per la maggior parte del tempo	1	2	3	4
17	Ho bisogno di conoscermi meglio per scegliere una professione	1	2	3	4
18	Mi è capitato di saltare i miei impegni scolastici o lavorativi perché sono stato fuori fino a tardi la sera precedente	1	2	3	4
19	Mi fido del mio giudizio	1	2	3	4
20	Il mio ambiente familiare a volte è davvero imprevedibile	1	2	3	4
21	Spesso mi verrebbe da prendere a pugni qualcuno	1	2	3	4
22	Penso che la mia pancia sia troppo grossa	1	2	3	4
~~23~~	~~In genere non vado molto d'accordo con le altre persone~~	~~1~~	~~2~~	~~3~~	~~4~~
24	Ho perso interesse nelle cose che mi hanno sempre divertito	1	2	3	4
25	Sono preoccupato perché non riesco a trovare una professione che mi interessi	1	2	3	4
26	Quando bevo a volte faccio cose che mi imbarazzano un po'	1	2	3	4
27	La mia famiglia cerca di gestire la mia vita	1	2	3	4

© 2011, C. Sica et al., *Q-PAD,* Trento, Erickson

28	Ho il terrore di aumentare di peso	1	2	3	4
29	Ultimamente ho avuto difficoltà a concentrarmi	1	2	3	4
30	Rimango deluso tutte le volte che do confidenza agli altri	1	2	3	4
31	Sono insoddisfatto perché non ho ancora progetti professionali per il futuro	1	2	3	4
32	Mi sono messo nei guai a causa del bere	1	2	3	4
33	Ultimamente sono spesso agitato	1	2	3	4
34	Le persone intorno a me non capiscono come sono veramente	1	2	3	4
35	Le cose sono andate di male in peggio	1	2	3	4
36	Sono preoccupato di non riuscire a trovare un corso di studi adatto a me	1	2	3	4
37	A volte usare alcol o altre sostanze mi aiuta a superare i miei problemi	1	2	3	4
38	I miei genitori non vogliono lasciarmi crescere	1	2	3	4
39	Se aumento di peso sono preoccupato di continuare ad aumentare	1	2	3	4
40	Spesso sono così nervoso che sento il mio cuore martellare	1	2	3	4
41	Sono spesso coinvolto in discussioni	1	2	3	4
42	Ultimamente il tempo non mi passa mai	1	2	3	4
43	Non so con quale criterio scegliere una professione	1	2	3	4
44	Non ho nessuna particolare potenzialità o talento	1	2	3	4
45	Mi sento soffocato dai miei genitori	1	2	3	4
46	Mi sento in forma	1	2	3	4
47	Mi preoccupo di cose di cui non si preoccupa la maggior parte delle persone	1	2	3	4
48	Pensieri tristi mi tengono sveglio la notte	1	2	3	4
49	Sebbene sappia che è arrivato il tempo per me di decidere, non sono ancora pronto per scegliere un'area di studio o una professione	1	2	3	4
50	Alcuni mi fanno notare il mio uso di alcol o di altre sostanze	1	2	3	4
51	Non mi sento capace come la maggior parte delle persone	1	2	3	4
52	La mia vita familiare è piacevole e soddisfacente	1	2	3	4
53	Ho fiducia in me stesso	1	2	3	4
54	Do troppa importanza al peso	1	2	3	4

© 2011, C. Sica et al., *Q-PAD,* Trento, Erickson

55	Penso di mostrare i segni di molto stress	1	2	3	4
56	Non vado d'accordo con i professori e le altre autorità	1	2	3	4
57	Dalle mie attività non ottengo lo stesso piacere di un tempo	1	2	3	4
58	Sento di essere forzato in una professione che non voglio	1	2	3	4
59	Le persone si sono approfittate di me mentre ero ubriaco o alticcio	1	2	3	4
60	Molto spesso agisco in modo da dovermi poi pentire	1	2	3	4
61	Mi godo davvero la vita	1	2	3	4
62	Sono assillato dal desiderio di essere più magro	1	2	3	4
63	Ultimamente le mie preoccupazioni hanno reso difficile addormentarmi	1	2	3	4
64	Sono stanco del modo in cui le persone mi trattano	1	2	3	4
65	Non mi importa quello che faccio, tanto le cose non miglioreranno	1	2	3	4
66	Sono ansioso perché mi è rimasto poco tempo per cercare una professione	1	2	3	4
67	Ho un'opinione molto positiva di me stesso	1	2	3	4
68	Non mi piace stare a casa perché nella mia famiglia si discute sempre	1	2	3	4
69	Mi sento spesso inquieto ma non so il perché	1	2	3	4
70	Sbaglio spesso nella scelta delle mie amicizie	1	2	3	4
71	Mi sembra di non potermi liberare dalla mia tristezza	1	2	3	4
72	I miei amici hanno un'idea migliore del loro futuro rispetto a quella che io ho del mio	1	2	3	4
73	Qualcuno dei miei amici non approva l'utilizzo che faccio dell'alcol o di altre sostanze	1	2	3	4
74	Le persone dicono che manco di fiducia in me stesso	1	2	3	4
75	Sono infastidito da pensieri da cui non riesco a liberarmi	1	2	3	4
76	Non ho fiducia nella maggior parte delle persone intorno a me	1	2	3	4
77	Sono molto indeciso sul mio futuro	1	2	3	4
78	A volte bere o usare altre sostanze mi mette nei guai o in situazioni pericolose	1	2	3	4
79	Mi sento frequentemente insoddisfatto della persona che sono	1	2	3	4
80	Ho paura dei conflitti con i miei genitori	1	2	3	4
81	Ho molta resistenza alla fatica	1	2	3	4

© 2011, G. Sica et al., Q-PAD, Trento, Erickson

EDI-3
Eating Disorder Inventory-3

David M. Garner
Adattamento italiano a cura di Marco Giannini, Linda Pannocchia, Riccardo Dalle Grave, Filippo Muratori e Valentina Viglione

Scrivi nome, data, età, sesso, stato civile e occupazione. Rispondi a tutte le domande contenute in questa pagina. Dopodiché, volta pagina e segui attentamente le istruzioni.

Nome ____________________ Cognome_____________ Data____/______/____

Età _____ Sesso ____ Stato civile__________ Occupazione________________

A. Peso attuale kg _____

B. Altezza cm _____

C. Qual è il peso più alto che hai mai raggiunto (escluse eventuali gravidanze)? Kg _____
Quanto tempo fa hai raggiunto questo peso per la prima volta? _____
Per quanto tempo hai mantenuto questo peso? _____

D. Qual è il peso più basso che hai raggiunto da adulto (o nell'adolescenza prima di 18 anni)? Kg _____
Quanto tempo fa hai raggiunto questo peso per la prima volta? _____ mesi fa
Per quanto tempo hai mantenuto questo peso _____ mesi

E. Quale peso hai mantenuto per il periodo di tempo più lungo? Kg _____
A che età hai raggiunto questo peso per la prima volta? _____ anni

F. Se il tuo peso è cambiato molto negli anni, c'è un peso al quale cerchi di ritornare quando non sei a dieta? □ Sì □ No
Se sì, qual è questo peso? Kg _____
A quale età hai raggiunto questo peso per la prima volta? _____ anni

G. Qual è il maggior numero di chili che hai mai perso? Kg _____
Li hai persi di proposito? □ Sì □ No
Che peso hai raggiunto dimagrendo? Kg _____
A che età hai raggiunto questo peso? _____ anni

H. Secondo te, quale sarebbe il tuo peso se non cercassi consapevolmente di controllarlo? Kg _____

I. Quanto ti piacerebbe pesare? Kg _____

J. A che età sono iniziati i problemi di peso (se ve ne sono)? _____ anni

K. Professione del padre ____

L. Professione della madre ____

Istruzioni

Le domande che seguono riguardano atteggiamenti, sentimenti e comportamenti relativi alla tua alimentazione e a te stesso/a.
Per ciascuna domanda decidi quale risposta corrisponde meglio a te scegliendo tra **A = SEMPRE, B = DI SOLITO, C = SPESSO, D = TALVOLTA, E = RARAMENTE, F = MAI**, e segnala con una crocetta. Ad esempio, se scegli la risposta SPESSO metti la crocetta sulla lettera C.
Rispondi a tutte le domande senza tralasciarne alcuna. **NON CANCELLARE!** Se vuoi cambiare la risposta, fai un cerchio intorno alla lettera sbagliata e metti la crocetta sulla lettera giusta.

A= SEMPRE; B= DI SOLITO; C=SPESSO; D= TALVOLTA; E= RARAMENTE; F=MAI

1.	Mangio dolci e carboidrati senza sentirmi nervoso/a	A	B	C	D	E	F
2.	Penso che il mio stomaco sia troppo grande	A	B	C	D	E	F
3.	Vorrei poter ritornare alla sicurezza dell'infanzia	A	B	C	D	E	F
4.	Mangio quando sono di cattivo umore	A	B	C	D	E	F
5.	Mi rimpinzo di cibo	A	B	C	D	E	F
6.	Vorrei essere più giovane	A	B	C	D	E	F
7.	Ho il pensiero della dieta	A	B	C	D	E	F
8.	Mi spavento quando i miei sentimenti sono troppo forti	A	B	C	D	E	F
9.	Penso che le mie cosce siano troppo grosse	A	B	C	D	E	F
10.	Mi sento una persona incapace	A	B	C	D	E	F
11.	Mi sento estremamente in colpa dopo avere mangiato troppo	A	B	C	D	E	F
12.	Penso che la grandezza del mio stomaco sia proprio giusta	A	B	C	D	E	F
13.	Nella mia famiglia sono accettati soltanto risultati eccellenti	A	B	C	D	E	F
14.	Il periodo più felice della vita è quando si è bambini	A	B	C	D	E	F
15.	Parlo dei miei sentimenti	A	B	C	D	E	F
16.	Ho il terrore di aumentare di peso	A	B	C	D	E	F
17.	Ho fiducia negli altri	A	B	C	D	E	F

18.	Mi sento solo/a al mondo	A	B	C	D	E	F
19.	Sono soddisfatto/a del mio aspetto fisico	A	B	C	D	E	F
20.	Generalmente sento le cose della mia vita sotto controllo	A	B	C	D	E	F
21.	Non riesco a capire chiaramente quale emozione sto provando	A	B	C	D	E	F
22.	Preferirei essere una persona adulta piuttosto che un/una bambino/a	A	B	C	D	E	F
23.	Riesco facilmente a comunicare con gli altri	A	B	C	D	E	F
24.	Vorrei essere qualcun altro	A	B	C	D	E	F
25.	Do troppa importanza al peso	A	B	C	D	E	F
26.	Riesco ad identificare chiaramente quale emozione sto provando	A	B	C	D	E	F
27.	Mi sento inadeguato/a	A	B	C	D	E	F
28.	Ho continuato ad abbuffarmi sentendo di non riuscire a fermarmi	A	B	C	D	E	F
29.	Da bambino/a facevo il possibile per non deludere i miei genitori e i miei insegnanti	A	B	C	D	E	F
30.	Ho rapporti stretti con gli altri	A	B	C	D	E	F
31.	Mi piace la forma del mio sedere	A	B	C	D	E	F
32.	Sono assillato/a dal desiderio di essere più magro/a	A	B	C	D	E	F
33.	Non so che cosa sta succedendo dentro di me	A	B	C	D	E	F
34.	Faccio fatica ad esprimere agli altri ciò che provo	A	B	C	D	E	F
35.	Il peso della vita adulta è troppo gravoso	A	B	C	D	E	F
36.	Non sopporto di non essere il/la migliore	A	B	C	D	E	F
37.	Mi sento sicuro/a di me stesso/a	A	B	C	D	E	F
38.	Ho il pensiero delle abbuffate	A	B	C	D	E	F
39.	Sono contento/a di non essere più un/una bambino/a	A	B	C	D	E	F

40.	Non riesco a capire se ho fame oppure no	A	B	C	D	E	F
41.	Ho una bassa stima di me stesso/a	A	B	C	D	E	F
42.	Sento di poter raggiungere le mete che mi prefiggo	A	B	C	D	E	F
43.	I miei genitori si aspettavano da me la perfezione	A	B	C	D	E	F
44.	Ho paura di perdere il controllo delle mie emozioni	A	B	C	D	E	F
45.	Penso che i miei fianchi siano troppo grandi	A	B	C	D	E	F
46.	Mangio moderatamente di fronte agli altri e mi rimpinzo quando sono andati via	A	B	C	D	E	F
47.	Mi sento gonfio/a dopo un pasto normale	A	B	C	D	E	F
48.	Penso che il periodo più felice sia quando si è bambini	A	B	C	D	E	F
49.	Se aumento di mezzo chilo sono preoccupato/a di continuare ad aumentare	A	B	C	D	E	F
50.	Sento di essere una persona che vale	A	B	C	D	E	F
51.	Quando sono di cattivo umore non so se sono triste, spaventato/a o arrabbiato/a	A	B	C	D	E	F
52.	Sento che devo fare le cose in maniera perfetta oppure non farle affatto	A	B	C	D	E	F
53.	Mi viene in mente di provare a vomitare per perdere peso	A	B	C	D	E	F
54.	Ho bisogno di tenere le persone ad una certa distanza (mi sento a disagio se qualcuno prova ad avvicinarsi troppo)	A	B	C	D	E	F
55.	Penso che la grandezza delle mie cosce sia giusta	A	B	C	D	E	F
56.	Mi sento vuoto/a emotivamente	A	B	C	D	E	F
57.	Riesco a parlare di ciò che penso e provo	A	B	C	D	E	F
58.	Il periodo migliore della vita è quando si diventa adulti	A	B	C	D	E	F
59.	Penso che il mio sedere sia troppo grande	A	B	C	D	E	F
60.	Provo sensazioni che non riesco bene a definire	A	B	C	D	E	F
61.	Mangio o bevo di nascosto	A	B	C	D	E	F

62.	Penso che i miei fianchi siano proprio della grandezza giusta	A	B	C	D	E	F
63.	Mi pongo obiettivi estremamente elevati	A	B	C	D	E	F
64.	Quando sono agitato/a o di cattivo umore ho paura di iniziare a mangiare	A	B	C	D	E	F
65.	Le persone a cui tengo veramente finiscono col deludermi	A	B	C	D	E	F
66.	Mi vergogno delle mie debolezze	A	B	C	D	E	F
67.	Gli altri potrebbero dire che sono emotivamente instabile	A	B	C	D	E	F
68.	Mi piacerebbe controllare completamente i bisogni del mio corpo	A	B	C	D	E	F
69.	Mi sento rilassato/a nella maggior parte delle situazioni di gruppo	A	B	C	D	E	F
70.	Dico impulsivamente cose che poi mi pento di avere detto	A	B	C	D	E	F
71.	Faccio di tutto per provare piacere	A	B	C	D	E	F
72.	Devo fare attenzione alla mia tendenza ad abusare di droghe	A	B	C	D	E	F
73.	Sono socievole con la maggior parte delle persone	A	B	C	D	E	F
74.	Mi sento intrappolato/a nei rapporti con gli altri	A	B	C	D	E	F
75.	L’astinenza mi fa sentire più forte spiritualmente	A	B	C	D	E	F
76.	Gli altri capiscono i miei veri problemi	A	B	C	D	E	F
77.	Non riesco a tenere lontano dalla mia mente pensieri strani	A	B	C	D	E	F
78.	Mangiare per il piacere è un segno di debolezza morale	A	B	C	D	E	F
79.	Sono incline a scatti di ira	A	B	C	D	E	F
80.	Credo che gli altri mi diano il credito che merito	A	B	C	D	E	F
81.	Devo stare attento/a alla mia tendenza ad abusare di alcolici	A	B	C	D	E	F
82.	Credo che rilassarsi sia semplicemente una perdita di tempo	A	B	C	D	E	F

83.	Gli altri potrebbero dire che mi irrito facilmente	A	B	C	D	E	F
84.	Mi sento perdente in ogni circostanza	A	B	C	D	E	F
85.	Ho forti sbalzi di umore	A	B	C	D	E	F
86.	Mi vergogno dei bisogni del mio corpo	A	B	C	D	E	F
87.	Preferisco trascorrere il tempo da solo/a piuttosto che con gli altri	A	B	C	D	E	F
88.	Le sofferenze rendono migliori	A	B	C	D	E	F
89.	So che la gente mi vuole bene	A	B	C	D	E	F
90.	Mi sento come se dovessi fare del male a me stesso/a o agli altri	A	B	C	D	E	F
91.	Penso di sapere realmente chi sono	A	B	C	D	E	F
